# Targeting the PTN/PTPRZ1-ROS Pathway to Promote Bone Regeneration

**DOI:** 10.3390/biomedicines13030695

**Published:** 2025-03-12

**Authors:** Kai Zhao, Yusi Guo, Ying He, Yujia Wu, Zhewen Hu, Xiaopei Chi, Xuliang Deng

**Affiliations:** 1 Department of Geriatric Dentistry, Peking University School and Hospital of Stomatology, Beijing 100081, China; zhaokaidentist@126.com (K.Z.); guoyusi@bjmu.edu.cn (Y.G.); heying@bjmu.edu.cn (Y.H.); kqwuyujia@bjmu.edu.cn (Y.W.); philip0323@163.com (Z.H.); chixiaopei851123@126.com (X.C.); 2 NMPA Key Laboratory for Dental Materials, National Engineering Research Center of Oral Biomaterials and Digital Medical Devices, Beijing Laboratory of Biomedical Materials, Beijing Key Laboratory of Digital Stomatology, Peking University School and Hospital of Stomatology, Beijing 100081, China

**Keywords:** reactive oxygen species, pleiotrophin, bone regeneration, stem cells, phosphorylation

## Abstract

**Background**: Osteoporosis is a global health problem that significantly decreases patients’ quality of life and causes tremendous medical burdens. Therefore, exploring effective targeting strategies for osteoporosis treatment is crucial. Previous studies have indicated that pleiotrophin (PTN) was a secretory factor involved in several biological processes, such as angiogenesis, neural development, and abnormal osteogenic functions in osteoporosis. However, the roles of PTN in osteogenics and the mechanisms remain unclear. **Methods**: In this study, we explored the effects and mechanisms of PTN in regulating osteogenic functions using real-time quantitative PCR, immunofluorescence, ALP detection, a TUNEL assay, RNA sequencing, and phosphorylation quantitative proteomics. Fracture-healing experiments in osteoporosis rats were also conducted to evaluate the osteogenic functions of PTN in vivo. **Results**: We found that PTN significantly inhibited apoptosis and promoted the osteogenic differentiation of rat bone marrow mesenchymal stem cells (rBMSCs). Further experiments showed that PTN regulated the biological functions of rBMSCs by promoting antioxidant functions and reducing cellular reactive oxygen species (ROS), thereby protecting rBMSCs from accumulated ROS. Additionally, we found that PTN binds to the PTPRZ1 receptor, inducing intracellular PLCG1 phosphorylation and NCOA3 nuclear translocation, which regulate the downstream antioxidant functions of rBMSCs. Additionally, we verified that PTN effectively promoted fracture healing in osteoporotic animals. **Conclusions**: This study elucidates the mechanisms by which PTN promotes osteogenesis and verifies this effect in vivo, offering an effective target for osteoporosis treatment.

## 1. Introduction

Osteoporosis is a systemic skeletal metabolic disease caused by multiple factors [1]. This disease is characterized by reduced bone mineral density and the structural degradation of bone tissue, resulting in poor mechanical properties and an increased risk of bone fracture [2]. Fractures caused by osteoporosis are increasingly common in middle-aged and older people, considerably decreasing their quality of life, creating high medical costs, and even significantly increasing mortality [3,4,5]. Therefore, osteoporosis has imposed enormous social and economic burdens and has become a global issue with extensive influences [1]. Currently, the treatment of osteoporosis relies mainly on drug therapy, including antiresorptive and anabolic drugs [6,7,8,9]. However, most drugs used in clinical practice have limitations such as poor targeting effects, unpredictable treatment results, and complications [10,11,12]. Therefore, exploring the pathogenesis of osteoporosis is important for identifying effective therapeutic targets.

Pleiotrophin (PTN), a secretory factor with essential biological functions, belongs to the heparin-binding family of proteins. The PTN sequence is highly conserved among different species and is involved in angiogenesis, neural system development, tumor regulation, epithelial cell differentiation, and other important biological processes [13,14,15,16,17]. However, the effects and mechanisms of action of PTN in bone metabolism remain unclear. A study employing microarray analysis revealed that PTN expression is significantly decreased in osteoblasts derived from patients with osteoporosis [18]. Another study investigating the relationships between osteoporosis and PTN genomic polymorphisms demonstrated that the promoter region polymorphisms of PTN were significantly associated with osteoporosis phenotypes in postmenopausal women [19]. However, the mechanisms underlying this phenomenon have not yet been elucidated. Therefore, it can be speculated that PTN participates in the pathogenesis of osteoporosis; however, the biological mechanisms remain unclear.

Bones are highly dynamic organs composed of multiple types of cells and an extracellular matrix organized in certain structures [20,21]. Abnormal metabolic homeostasis during bone remodeling is the basis for the pathogenesis of osteoporosis [22,23,24]. Bone marrow mesenchymal stem cells (BMSCs) are cells with multidirectional differentiation potential and are located in the bone marrow. The differentiation of BMSCs is closely related to bone metabolic homeostasis, which is regulated by genetic, epigenetic, and physical or chemical factors [25,26,27]. Reactive oxygen species (ROS) include both free radical and non-free radical species, such as superoxide (O_2_^•−^), hydroxyl radicals (HO^•^), and hydrogen peroxide (H_2_O_2_). ROS are products of redox processes during cellular metabolism and are precisely regulated. An imbalance in redox homeostasis resulting from internal and external factors can lead to excessive ROS accumulation and pathological changes [28,29,30]. Additionally, ROS can lead to the structural destruction and functional damage of proteins, membrane lipids, nucleic acids, and other macromolecules, further inducing cell apoptosis, senescence, and functional degeneration [31,32,33]. Moreover, previous studies have suggested that ROS play a role in the pathogenesis of diseases with abnormal bone metabolism [34,35].

In this study, we explored the effects of PTN on the biological functions of BMSCs. We found that PTN promoted antioxidant function and reduced cellular ROS levels, thereby inhibiting cell apoptosis and promoting bone regeneration. The downstream mechanism was further analyzed, and the osteogenesis-promoting effect of PTN was verified in vivo. This study identifies a potential target for the clinical treatment of osteoporosis.

## 2. Materials and Methods

### 2.1. Cell Culture

Rat BMSCs (rBMSCs) were purchased from Cyagen Biosciences (Guangzhou, China; RASMX-01001). Cells were cultured in α-minimum essential medium (α-MEM, Gibco, Grand Island, NY, USA) with 10% fetal bovine serum (FBS, Gibco, USA) and 1% antibiotics (streptomycin–penicillin, Gibco, USA). The temperature of the cell incubator was set at 37 °C with 5% CO_2_. The medium was refreshed every 2–3 days until the confluence reached 90%.

### 2.2. Real-Time Quantitative PCR (qPCR)

Total RNA was extracted from rBMSCs using Total RNA Extraction Reagent (Yeasen, Shanghai, China, 10606ES60) following the manufacturer’s protocol. Reverse transcription was conducted using the PrimeScript Master Mix (TaKaRa, Osaka, Japan). Subsequently, qPCR was performed using the Universal SYBR Green Mix (Abclonal, Wuhan, China, #RK21203) on a real-time PCR Detection System (Bio-Rad, Hercules, CA, USA). GAPDH was used as an internal reference to calculate the relative expression levels. The primers used for qPCR are listed in Table S1.

### 2.3. Immunofluorescence

For immunofluorescence, rBMSCs were fixed with 4% paraformaldehyde (PFA) at room temperature for 15 min. The samples were permeabilized with 0.1% Triton X-100 (Sigma-Aldrich, Shanghai, China) for 10 min and blocked with 3% bovine serum albumin for 1 h. Subsequently, the samples were incubated with specific primary antibodies at 4 °C overnight and washed three times using PBS. The samples were incubated with fluorochrome-conjugated secondary antibodies at room temperature for 1 h and washed three times using PBS. The nuclei were stained with DAPI, and fluorescence images were obtained using a Leica TCS SP8 microscope (Leica, Wetzlar, Germany). The NCOA3 inhibitor, SI-2 (MedChemExpress, Monmouth Junction, NJ, USA, HY-101447), was used for the NCOA3 inhibition experiment. Rabbit anti-PTPRZ1 antibody (1:200, Proteintech, Chicago, IL, USA, 55125-1-AP) was used for neutralizing antibody blocking experiments, and rabbit IgG antibody (1:200, Proteintech, Chicago, IL, USA, 30000-0-AP) was used as a control.

The antibodies used in this study were as follows: mouse anti-BMP2 (1:500, Proteintech, 66383-1-Ig); rabbit anti-OCN (1:500, Proteintech, 23418-1-AP); rabbit anti-Caspase-3 (1:400, Proteintech, 19677-1-AP); rabbit anti-Caspase-8 (1:400, Proteintech, 13423-1-AP); rabbit anti-GLRX (1:500, Proteintech, 15804-1-AP); rabbit anti-GPX3 (1:500, Proteintech, 13947-1-AP); rabbit anti-PRDX6 (1:500, Proteintech, 13585-1-AP); rabbit Phospho-PLCG1 (Tyr1253) antibody (1:500, GeneTex, Irvine, CA, USA, GTX32238); rabbit Phospho-NCOA3 (Ser214) antibody (customized from QYAOBIO, Shanghai, China); goat Anti-Mouse (Alexa Fluor^®^ 488) secondary antibody (1:400, Abcam, Cambridge, UK, ab150113); and goat Anti-Rabbit (Alexa Fluor^®^ 488) secondary antibody (1:400, Abcam, Cambridge, UK, ab150077).

### 2.4. ALP Staining and Quantification Analysis

The rBMSCs were seeded in a 24-well plate. For ALP staining, the rBMSCs were washed with PBS after induction and fixed with 4% paraformaldehyde at room temperature for 15 min. ALP staining was performed using a BCIP/NBT Alkaline color development kit (Beyotime, Shanghai, China, C3206) following the manufacturer’s protocol. ALP was quantified using an ALP assay kit (Beyotime, Shanghai, China, P0321M) following the manufacturer’s protocol.

### 2.5. TUNEL Assay and DAG Detection

Terminal deoxynucleotidyl transferase-mediated nick-end labeling (TUNEL) experiments were conducted using the TUNEL Apoptosis Detection Kit (Yeasen, China, YSFluorTM 488), according to the manufacturer’s protocol. Briefly, rBMSCs were fixed with 4% paraformaldehyde at room temperature for 15 min and permeabilized with 0.1% Triton X-100 for 10 min. The rBMSCs were subsequently incubated with labeling buffers at 37 °C away from light for 60 min. DAPI was used for nuclear staining, and fluorescence images were captured using a laser confocal microscope (Leica, Wetzlar, Germany, TCS SP8). Diacylglycerol (DAG) levels in the samples were detected using a Diacylglycerol Assay Kit (Abcam, ab242293) according to the manufacturer’s protocol.

### 2.6. RNA Sequencing

RNA was extracted from rBMSCs using RNA Extraction Reagent (Yeasen, China, 10606ES60) following the manufacturer’s protocol. The RNA quality was evaluated using a Nanodrop OneC spectrophotometer (Thermo Fisher Scientific, Waltham, MA, USA) and an Agilent 2100 Bioanalyzer (Agilent Technologies Inc., Santa Clara, CA, USA). After library construction, RNA sequencing was performed using the Illumina NextSeq 500 platform (Illumina, San Diego, CA, USA). Differentially expressed genes (DEGs) between the groups were analyzed using DESeq2 (R4.1.2). A padj cutoff of 0.05 and a log_2_FoldChange cutoff of 1 were used to judge the statistically significant differences between the groups. Gene function enrichment analysis was performed using the Kyoto Encyclopedia of Genes and Genomes (KEGG) and Gene Ontology (GO) databases. The functional conditions of the DEGs between the groups were analyzed using gene set enrichment analysis (GSEA).

### 2.7. ROS Staining

ROS staining was performed using the fluorescent probe DCFH-DA. Briefly, rBMSCs were incubated with DCFH-DA (10 μm) at 37 °C away from light for 45 min. Post-incubation, rBMSCs were washed three times with PBS. DAPI was used for nuclear staining, and the ROS staining was observed using a laser confocal microscope (Leica, Wetzlar, Germany, TCS SP8).

### 2.8. Four-Dimensional Label-Free Phosphorylation Quantitative Proteomic

The samples were added to a lysate buffer containing protease inhibitor (Sangon Biotech, Shanghai, China, A610425-0005) and phosphatase inhibitor (Roche, Basel, Switzerland, 4906837001). After ultrasonic crushing and centrifugation, the sample supernatants were collected. The protein concentration of each sample was determined using the bicinchoninic acid method. Following digestion and enzymolysis, the peptides were desalted using a Sep-Pak C18 cartridge, and the phosphorylated peptides were enriched and labeled. The samples were analyzed using an EASY-nLC 1200 liquid chromatograph (Thermo Fisher Scientific,, Waltham, MA, USA). MaxQuant version (v1.5.2.8) was used for data processing with FDR < 0.01 for protein, peptides, and modifications. Annotation and functional analysis of the identified phosphorylated peptides and proteins were mainly based on the UniProt, GO, KEGG, InterPro, and Reactome databases. GSEA was conducted for the functional enrichment analysis of differentially phosphorylated proteins.

### 2.9. Animal Experiments

Seven-week-old Sprague Dawley female rats were obtained from Charles River Labs (Beijing, China). All the animals were maintained in a pathogen-free environment and allowed free access to food and water throughout the experiment. At the end of the animal study, the animals were humanely sacrificed, and their organs were collected for research. Power calculation was performed for sample size determination to ensure the power of the test. The experimental animals were randomly grouped by the random number table method. Animal experiment operators were not informed of grouping assignments to ensure the blinding procedure. Osteoporosis rat models were constructed using ovariectomy, following previous protocols [36,37]. Mid-diaphyseal femoral fractures were created according to previously described methods [38,39]. A 0.5 mg/kg body weight recombinant rat pleiotrophin (CUSABIO, Wuhan, China, CSB-EP019009RA) or vehicle was injected into the fracture area 3 days post-surgery. The drugs were then injected every 3 days for a total of five doses. Animal femur samples were collected during weeks 3 and 6 post-surgery. Appropriate anesthesia and fine operation were performed to reduce surgical trauma and relieve pain for animals. A good feeding environment with adequate food and water was provided after the operation.

### 2.10. Micro-CT Evaluation

Bone samples were scanned using a micro-CT scanner (μCT 100; Scanco Medical, Wangen-Bruttisellen, Switzerland) with a voxel resolution of 20 μm, 300 ms integration time, 80 kVP peak voltage, and 200 mA current. Three-dimensional histomorphometric analysis was conducted to assess fracture healing. Quantitative parameters, including the bone tissue/total tissue volume ratio (BV/TV), bone mineral density (BMD), trabecular thickness (Tb. Th), and trabecular distance (Tb. Sp) were calculated to evaluate fracture healing following the algorithm from the manufacturer.

### 2.11. Histological Assay

The surrounding soft tissue of the healing femurs was carefully dissected and fixed in 4% PFA for 24 h. Bone specimens were decalcified with 15% EDTA, with daily changes in the decalcifying fluid for 25 days. The decalcified bone tissue was dehydrated using an ethanol concentration gradient, permeabilized with xylene, and embedded in paraffin. The tissue was sliced at a 6 µm thickness and stained with hematoxylin and eosin (HE) and Masson staining. Fracture healing was evaluated using an optical microscope. The proportions of new bone after Masson staining were quantitatively analyzed using ImageJ software (ImageJ 1.45, NIH, Bethesda, MD, USA).

### 2.12. Statistical Analysis

The data are presented as the means ± SEMs (standard errors of the means). The normality of the data was tested using the Shapiro–Wilk test. Statistical differences between samples of the two groups were analyzed using Student’s *t*-test, whereas differences among samples from more than two groups were analyzed using one-way ANOVA followed by Tukey’s post hoc test. Statistical significance was set at *p* < 0.05. Statistical analyses were performed using SPSS version 22.0 (IBM Corp., Armonk, NY, USA) and GraphPad Prism version 7.2.0 software (GraphPad Software, Inc., San Diego, CA, USA).

## 3. Results

### 3.1. PTN Promotes Osteogenic Differentiation and Effectively Inhibits Apoptosis of rBMSCs

**Figure 1:**
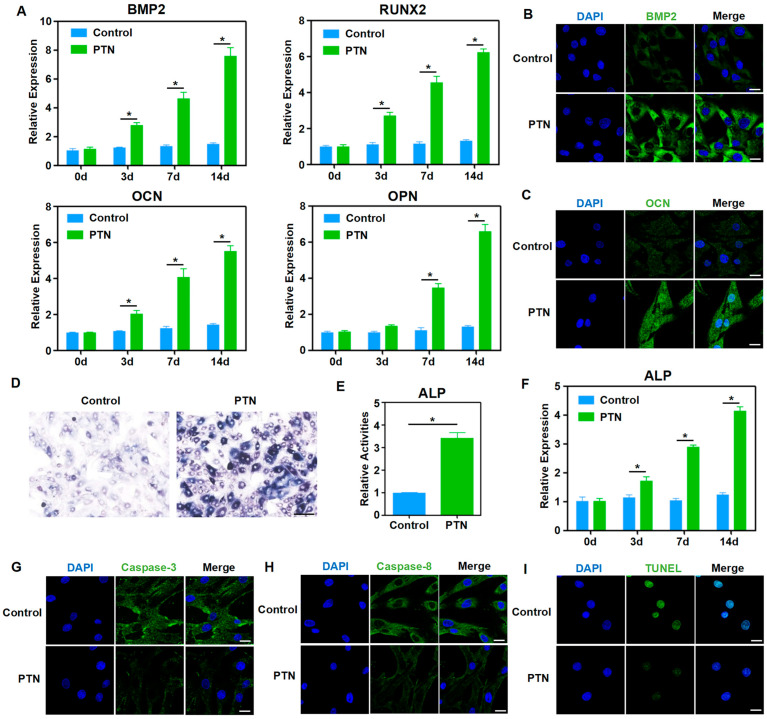
PTN promoted osteogenic functions and inhibited the apoptosis of rBMSCs. (**A**) The mRNA levels of osteoblastic markers (BMP2, RUNX2, OCN, and OPN) in rBMSCs were detected using qPCR at 3 d, 7 d, and 14 d after induction with PTN. (**B**,**C**) The expression levels of BMP2 (**B**) and OCN (**C**) were detected via immunofluorescence staining 7 d after PTN induction. Scale bar: 20 μm. (**D**,**E**) The activities of ALP in rBMSCs were measured qualitatively (**D**) and quantitatively (**E**) 7 days after induction with PTN. Scale bar: 100 μm (**D**). (**F**) The mRNA levels of ALP in rBMSCs were detected using qPCR at 3 d, 7 d, and 14 d after induction with PTN. (**G**,**H**) The expressions of Caspase-3 (**G**) and Caspase-8 (**H**) were detected through immunofluorescence staining 7 d after PTN induction. Scale bar: 20 μm. (**I**) The apoptosis levels of rBMSCs were detected through TUNEL staining 7 days after PTN induction. Scale bar: 20 μm. Each group contained at least three biological replicates (**A**,**E**,**F**). Statistically significant differences between the groups were analyzed using Student’s *t*-test (**E**) or one-way ANOVA followed by Tukey’s post hoc test (**A**,**F**). * *p* < 0.05.

Fate determination and rBMSC regulation play essential roles in bone metabolism and regeneration; therefore, we evaluated the biological effects of PTN induction on rBMSCs. Our findings showed that PTN significantly increased the mRNA levels of several osteogenic markers, including BMP2, RUNX2, OCN, and OPN, as determined through qPCR analysis (Figure 1A). Immunofluorescence staining of BMP2 and OCN revealed that PTN induction markedly increased BMP2 and OCN protein levels (Figure 1B,C). Additionally, PTN induction significantly elevated ALP activity and its corresponding mRNA levels (Figure 1D–F). These results indicated that PTN effectively promoted the osteogenic differentiation of rBMSCs. Immunofluorescence staining of Caspase-3 and Caspase-8 showed that PTN markedly decreased the levels of these apoptosis-related proteins (Figure 1G,H). Additionally, TUNEL staining confirmed that PTN significantly inhibited apoptosis (Figure 1I). These findings suggest that PTN could effectively promote osteogenic differentiation and inhibit the apoptosis of rBMSCs.

### 3.2. PTN Regulates the Biological Behavior of rBMSCs by Promoting Antioxidant Functions and Decreasing ROS

**Figure 2:**
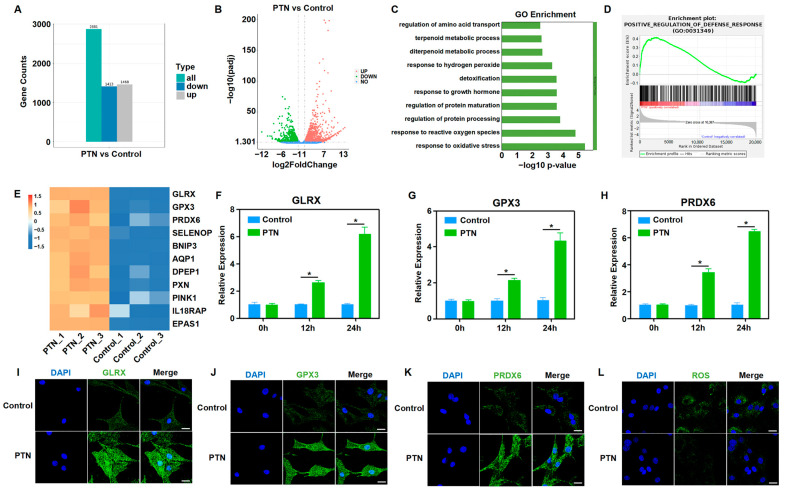
PTN regulated the biological behavior of rBMSCs by promoting antioxidant functions and decreasing cellular ROS. (**A**,**B**) RNA-seq was performed to analyze the gene expression conditions of rBMSCs stimulated using PTN. PTN significantly induced transcriptome profile changes, as shown in the histogram (**A**) and volcano diagram (**B**). (n = 3). (**C**,**D**) GO (**C**) and GSEA (**D**) showed that oxidative stress regulation and cellular defense response function were significantly enriched in rBMSCs after PTN stimulation. (**E**) Gene differential expression analysis demonstrated that PTN significantly promoted the expression of antioxidant factors in rBMSCs, as shown in the heat map. (**F**–**H**) The mRNA levels of antioxidant factors (GLRX, GPX3, and PRDX6) in rBMSCs were detected using qPCR at 12 h and 24 h after induction with PTN. (**I**–**K**) The expression levels of GLRX (**I**), GPX3 (**J**), and PRDX6 (**K**) were detected through immunofluorescence staining 24 h after PTN induction. Scale bar: 20 μm. (**L**) The cellular ROS levels in the rBMSCs were detected 24 h after PTN induction. Scale bar: 20 μm. Each group contained at least three biological replicates (**F**–**H**). Significant differences between the groups were analyzed using one-way ANOVA followed by Tukey’s post hoc test. * *p* < 0.05.

To further clarify the mechanisms underlying the regulation of rBMSC function by PTN, we performed RNA sequencing and bioinformatics analyses after PTN induction. General analysis showed that the transcription profiles of rBMSCs changed significantly following PTN induction (Figure 2A,B). GO functional and GESA enrichment analyses revealed that PTN-treated rBMSCs were significantly enriched in oxidative stress regulation and cellular defense response functions (Figure 2C,D). As shown in the heat map, PTN stimulation significantly increased the expression of antioxidant factors, including GLRX, GPX3, and PRDX6 (Figure 2E). We also verified the effects of PTN stimulation on antioxidant factors (GLRX, GPX3, and PRDX6) using qPCR and immunofluorescence staining (Figure 2F–K). Additionally, PTN induction significantly inhibited ROS responses in rBMSCs (Figure 2L). Our findings confirm that PTN regulates the biological functions of rBMSCs by promoting the expression of antioxidant factors and decreasing ROS levels.

### 3.3. PTN-Regulated rBMSCs Function Through the PTPRZ1 Receptor

**Figure 3:**
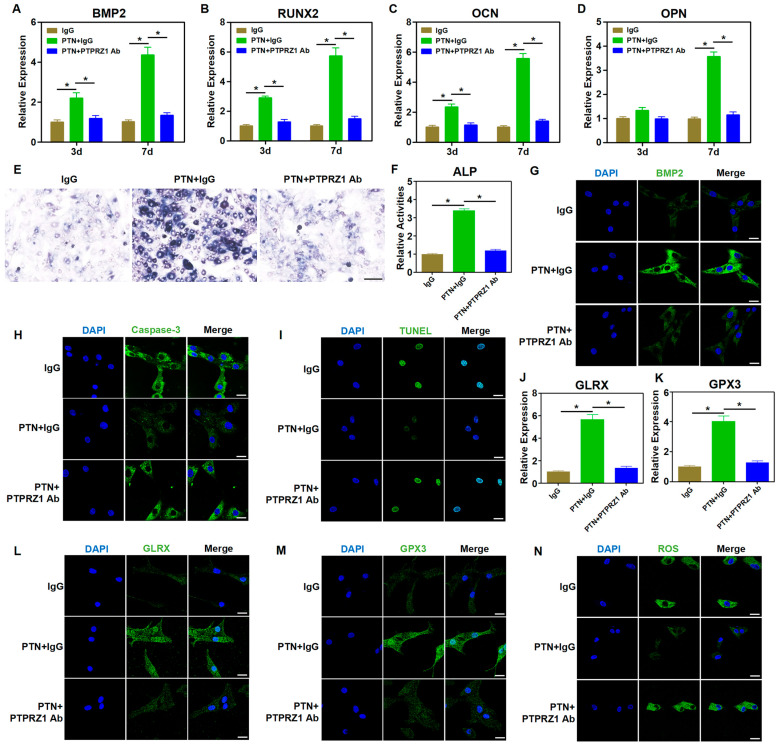
PTN regulated the biological behavior of rBMSCs through the PTPRZ1 receptor. (**A**–**D**) The mRNA levels of osteoblastic markers (BMP2, RUNX2, OCN, and OPN) in rBMSCs were detected through qPCR at 3 d and 7 d after induction. The neutralizing antibody of PTPRZ1 was used to block PTN binding with PTPRZ1 with IgG as a control. (**E**,**F**) The activities of ALP in rBMSCs were detected qualitatively (**E**) and quantitatively (**F**) 7 days after induction. Scale bar: 100 μm (**E**). (**G**) The expression level of BMP2 was detected via immunofluorescence staining 7 d after induction. Scale bar: 20 μm. (**H**) The expression level of Caspase-3 was detected via immunofluorescence staining 7 d after induction. Scale bar: 20 μm. (**I**) The apoptosis levels of rBMSCs were detected through TUNEL staining 7 days after induction. Scale bar: 20 μm. (**J**,**K**) The mRNA levels of GLRX (**J**) and GPX3 (**K**) in rBMSCs were detected through qPCR 24 h after induction. (**L**,**M**) The expression levels of GLRX (**L**) and GPX3 (**M**) were detected through immunofluorescence staining 24 h after induction. Scale bar: 20 μm. (**N**) The cellular ROS levels in the rBMSCs were detected 24 h after induction. Scale bar: 20 μm. Each group contained at least three biological replicates (**A**–**D**,**F**,**J**,**K**). Significant differences between the groups were analyzed using one-way ANOVA followed by Tukey’s post hoc test. * *p* < 0.05.

As an exocrine factor, PTN regulates cellular functions by binding to membrane receptors and activating downstream signaling pathways [40]. Previous studies have suggested that PTPRZ1, a membrane receptor of PTN, is involved in osteogenic functions [41,42]. We hypothesized that PTN might regulate the biological functions of rBMSCs through PTPRZ1. A neutralizing antibody against PTPRZ1 (anti-PTPRZ1) was used to block PTN binding to the PTPRZ1 receptor, and its downstream effects were explored. Blocking the binding of PTN to PTPRZ1 using a neutralizing antibody significantly inhibited the PTN-induced expression of osteogenic markers (Figure 3A–D,G and Figure S2). Additionally, blocking PTN and PTPRZ1 binding significantly inhibited the promotion of PTN-induced ALP activity (Figure 3E,F and Figure S1). These findings confirmed that PTN promotes the osteogenic function of rBMSCs through the receptor PTPRZ1. Immunofluorescence of apoptosis-related proteins and TUNEL staining showed that anti-PTPRZ1 inhibited PTN-induced apoptosis (Figure 3H,I and Figure S3). Furthermore, anti-PTPRZ1 significantly inhibited the regulation of antioxidant factors (GLRX, GPX3, and PRDX6) and ROS response induced with PTN (Figure 3J–N and Figure S4). Our study findings establish that PTN regulated the biological functions of rBMSCs by binding to the PTPRZ1 receptor.

### 3.4. PTN Regulates Antioxidant Functions Through PLCG1 Phosphorylation and NCOA3 Nucleus Translocation

**Figure 4:**
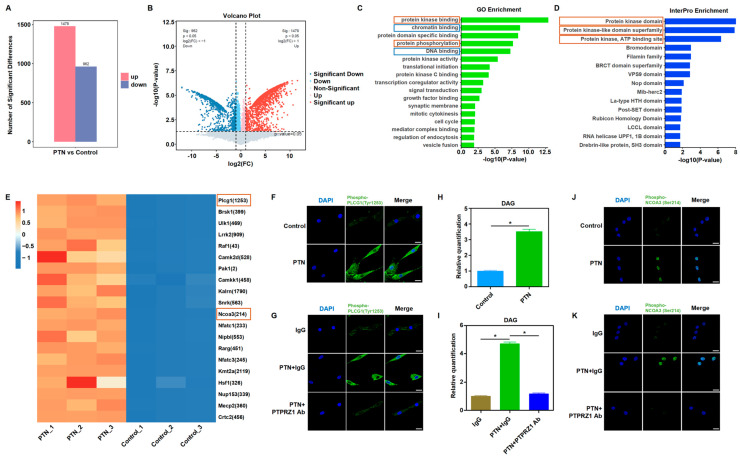
PTN-induced PLCG1 phosphorylation and NCOA3 nucleus translocation. (**A**,**B**) High-throughput mass spectrometry was performed to explore the phosphate homeostasis changes after PTN induction. PTN significantly induced phosphorylation profile changes, as shown in the histogram (**A**) and volcano diagram (**B**). (n = 3). (**C**,**D**) GO (**C**) and InterPro (**D**) enrichment analysis showed that rBMSCs were significantly enriched in kinase activities, phosphorylation modification functions, and epigenetic regulations after PTN stimulation. (**E**) Differential analysis of phosphorylation modifications revealed that PTN significantly promoted PLCG1 (Tyr1253) and NCOA3 (Ser214) phosphorylation in rBMSCs, as shown in the heat map. (**F**,**G**) PLCG1 (Tyr1253) phosphorylation conditions were detected by immunofluorescence staining after PTN induction (**F**) or anti-PTPRZ1 blocking (**G**). Scale bar: 20 μm. (**H**,**I**) DAG levels in rBMSCs were detected after PTN induction (**H**) or anti-PTPRZ1 blocking (**I**). (**J**,**K**) PTN promoted phosphorylated NCOA3 nucleus translocation detected through immunofluorescence staining (**J**), and this effect was blocked by anti-PTPRZ1 treatment (**K**). Scale bar: 20 μm. Each group contained at least three biological replicates (**H**,**I**). The significance of differences between the groups was analyzed using Student’s *t*-test (**H**) or one-way ANOVA followed by Tukey’s post hoc test (**I**). * *p* < 0.05.

We further explored the cascade signaling pathway that mediates PTN/PTPRZ1 and its downstream effects. The biological activity of PTPRZ1, a protein tyrosine phosphatase, changes after binding with PTN [13,15]. We speculated that PTN/PTPRZ1 binding may induce changes in cellular phosphate homeostasis. Using high-throughput phosphorylation mass spectrometry, we investigated the changes in phosphate homeostasis after PTN induction. The data showed good consistency within the PTN and control groups, with uniform expression levels across the six groups (Figure S5). PTN induction significantly altered the cellular phosphorylation profiles (Figure 4A,B). GO and InterPro enrichment analyses revealed that rBMSCs were significantly enriched in the kinase activities, phosphorylation modification, and epigenetic regulation, suggesting that PTN modulates cellular phosphorylation and epigenetic functions to produce downstream biological effects (Figure 4C,D). Bioinformatic analysis of the mass spectrometry data showed that PTN significantly induced PLCG1 (Tyr1253) phosphorylation (Figure 4E). Immunofluorescence analysis validated the phosphorylation of PLCG1 (Tyr1253) following PTN induction (Figure 4F,G). Previous studies have demonstrated that phosphorylation of PLCG1 promotes its biological activity, increases DAG levels, and activates PKC [43,44,45]. Our findings verify that PTN induction increased DAG levels, and these effects were blocked by the PTPRZ1 neutralizing antibody (Figure 4H,I). Previous studies have shown that PKC induces the phosphorylation and nucleation of NCOA3 and promotes the function of NRF2, an important antioxidant transcription factor [46,47,48]. Mass spectrometry analysis revealed increased phosphorylation of NCOA3 (Ser214) after PTN induction (Figure 4E). Additionally, we verified the nuclear translocation of phosphorylated NCOA3 (Figure 4J,K).

**Figure 5:**
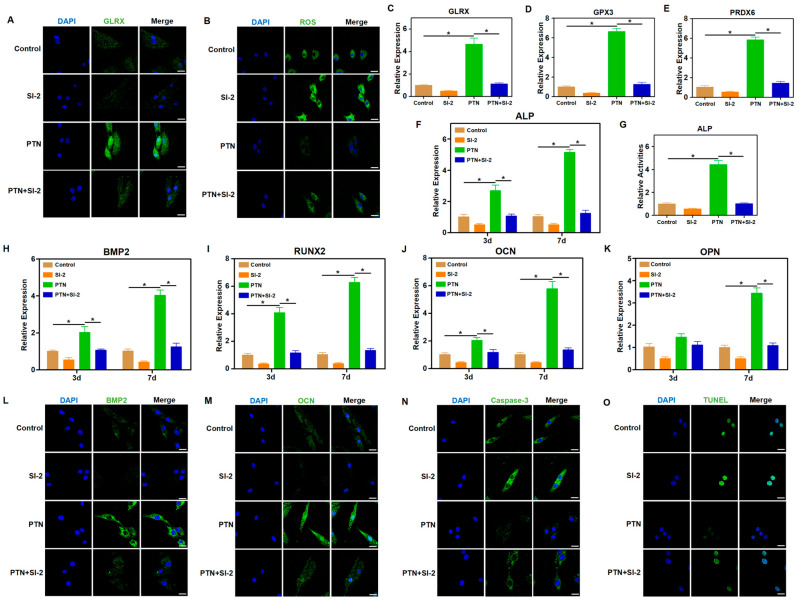
NCOA3 mediated the regulatory effects of PTN on rBMSCs. (**A**) The expression level of GLRX was detected via immunofluorescence staining 24 h after induction. SI-2 (NCOA3 inhibitor) was used to block the downstream effects of PTN induction. Scale bar: 20 μm. (**B**) Cellular ROS levels in rBMSCs were detected 24 h after treatment. Scale bar: 20 μm. (**C**–**E**) The mRNA levels of GLRX (**C**), GPX3 (**D**), and PRDX6 (**E**) in rBMSCs were detected through qPCR 24 h after induction. (**F**) The mRNA level of ALP in rBMSCs was detected through qPCR at 3 d and 7 d after induction. (**G**) The activities of ALP in rBMSCs were detected quantitatively 7 days after induction. (**H**–**K**) The mRNA levels of BMP2 (**H**), RUNX2 (**I**), OCN (**J**), and OPN (**K**) in rBMSCs were detected through qPCR at 3 d and 7 d after induction. (**L**,**M**) The expression levels of BMP2 (**L**) and OCN (**M**) were detected through immunofluorescence staining 7 d after induction. Scale bar: 20 μm. (**N**) The expression level of Caspase-3 was detected via immunofluorescence staining 7 d after induction. Scale bar: 20 μm. (**O**) The apoptosis levels of rBMSCs were detected through TUNEL staining 7 days after induction. Scale bar: 20 μm. Each group contained at least three biological replicates (**C**–**K**). Significant differences between the groups were analyzed using one-way ANOVA followed by Tukey’s post hoc test. * *p* < 0.05.

To confirm that NCOA3 acts downstream of PTN/PTPRZ1 to influence cellular functions, we used SI-2, an NCOA3 inhibitor, and tested its downstream functions. NCOA3 inhibition significantly blocked the PTN-induced regulation of antioxidant factors and ROS responses (Figure 5A–E, Figures S6 and S7). Additionally, NCOA3 inhibition blocked a PTN-induced increase in osteogenic markers and ALP activity (Figure 5F–M). Immunofluorescence and TUNEL staining for Caspase-3 and Caspase-8 revealed that NCOA3 inhibition significantly blocked PTN-mediated cell apoptosis regulation (Figure 5N,O and Figure S8). Our findings indicate that PTN regulates the biological functions of rBMSCs through PLCG1 phosphorylation and NCOA3 nucleus translocation.

### 3.5. PTN Promotes Fracture Healing in Osteoporosis Rats

**Figure 6:**
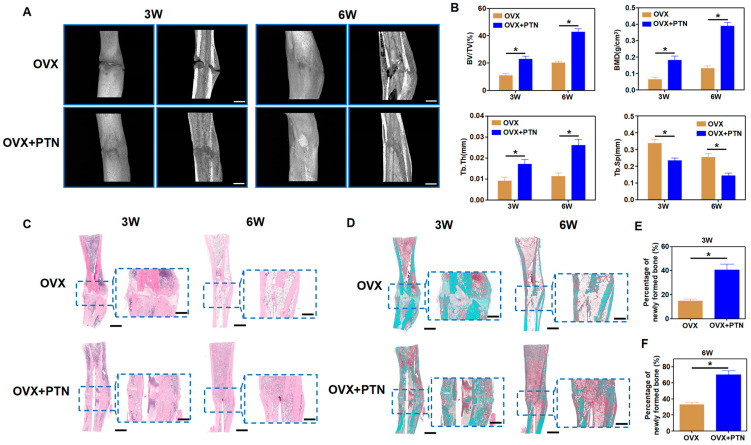
PTN promoted fracture healing in osteoporosis animals. (**A**) PTN promoted fracture healing in osteoporotic rats in the third and sixth weeks, as shown by micro-CT scanning. Scale bar: 2 mm. (**B**) Quantitative analysis of micro-CT scanning parameters, including BV/TV, BMD, Tb. Th, and Tb. Sp. n = 6. (**C**) Fracture healing in osteoporotic rats was evaluated through HE staining of bone tissue in the third and sixth weeks. Scale bar: 2 mm (outer). Scale bar: 1 mm (inner). (**D**) Fracture healing in osteoporotic rats was evaluated through Masson staining in the third and sixth weeks. Scale bar: 2 mm (outer). Scale bar: 1 mm (inner). (**E**,**F**) Quantitative analysis of Masson staining showed the percentages of newly formed bones in the third week (**E**) and sixth week (**F**). n = 6. Statistically significant differences between the groups were analyzed using Student’s *t*-test (**E**,**F**) or one-way ANOVA (**B**) followed by Tukey’s post hoc test. * *p* < 0.05.

**Figure 7:**
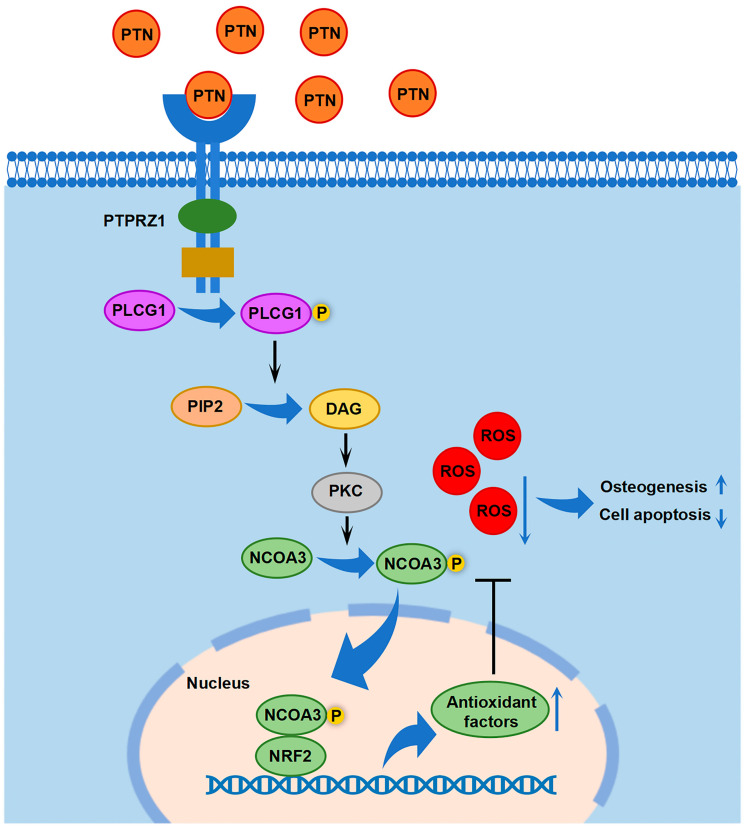
Schematic diagram showing the downstream cascade signaling by which PTN regulates rBMSC functions.

To verify the osteogenic effects of PTN in vivo, we established an osteoporotic rat model via ovariectomy and evaluated the effects of PTN on fracture healing. Micro-CT scanning showed that PTN significantly promoted fracture healing in osteoporotic rats both in the third and sixth weeks (Figure 6A). Quantitative analysis using micro-CT confirmed these effects (Figure 6B). Additionally, HE and Masson staining showed that bone healing and new bone formation in the PTN groups were significantly superior to those of the control groups (Figure 6C–F). Animal experiments were conducted to confirm whether PTN promotes fracture healing in osteoporotic animals. Our findings revealed that PTN promoted osteogenic differentiation and inhibited apoptosis. We clarified that PTN exerts its effects by inducing PLCG1 (Tyr1253) phosphorylation and NCOA3 nuclear translocation through the PTPRZ1 receptor, promoting the expression of antioxidant factors, and decreasing cellular ROS (Figure 7).

## 4. Discussion

Osteoporosis is a public health problem that affects the health and quality of life of individuals worldwide. As the population ages, the social problems and medical burdens caused by osteoporosis are expected to further intensify [49]. Therefore, exploring effective methods to relieve or cure osteoporosis is of great clinical significance [1]. In this study, we found that PTN promotes the osteogenic differentiation of rBMSCs and bone formation by regulating antioxidant factors. Additionally, the osteogenesis-promoting effects of PTN were verified in vivo. Thus, this study provides a potential strategy for the clinical treatment of osteoporosis.

Appropriate medication intake is recommended for the management of osteoporosis and its complications, in addition to improving patients’ lifestyle habits, such as reducing alcohol consumption, quitting smoking, engaging in moderate exercise, maintaining a balanced diet, and vitamin D supplementation [6,7]. Currently, several drugs are prescribed for the treatment of osteoporosis, including bisphosphonates, denosumab, selective estrogen receptor modulators, mixed steroid receptor agonists, teriparatide, and PTHrP analogs [8,9]. Bisphosphonates and denosumab are drugs commonly used for the clinical treatment of osteoporosis. Both drugs can inhibit the activities and functions of osteoclasts and, therefore, inhibit bone resorption for the treatment of osteoporosis [50,51]. Clinical results have shown that both bisphosphonates and denosumab can improve bone mineral density and reduce the incidence of bone fractures to a certain extent; however, individual differences exist in the therapeutic effects of both drugs [52,53]. Additionally, side effects and complications of these two drugs have been reported, including rash, ocular validation, joint and muscle pain, osteonecrosis of the jaw, and atypical femoral fractures [10,11,12]. Our study revealed that PTN may be a potential therapeutic target in osteoporosis. PTN induces the expression of intracellular antioxidant factors, reduces cellular ROS, and protects cells from injury. A limitation of this study is that no experiment was conducted to compare the treatment effects of PTN with other drugs for osteoporosis. It is of great significance to perform comparative studies that explicitly show the treatment differences of PTN with bisphosphonates, denosumab, or other drugs. We look forward to future studies to clarify these problems. PTN, as a secretory factor, might be dose dependent. However, only the effects of a single dose (0.5 mg/kg) were explored through animal experiments in our study. This is another limitation of this study. Future studies should be performed to clarify the dose-dependent effect of PTN. In this study, the osteogenic function of PTN was verified through a fracture healing experiment in rats with a follow-up period of 3 weeks and 6 weeks. Future studies should be conducted in large animals with longer follow-up periods to provide more powerful evidence for the clinical application of PTN. Previous studies have shown that PTN plays an important role in angiogenesis and neural development [14,16]. Therefore, we speculate that PTN promotes the fracture healing process not only by increasing the osteogenic differentiation of BMSCs but also by promoting angiogenesis and nerve regeneration in the fracture area. Thus, PTN may promote bone tissue regeneration through multiple biological mechanisms, including osteogenesis, angiogenesis, and neurogenesis. However, this hypothesis warrants further investigation. PTN could promote angiogenesis and oncogenesis reported in previous studies [13,14,15,16,17]. Therefore, there are risks of ectopic blood vessel formation and tumorigenesis during the application of PTN to promote bone fracture healing. We are looking forward to further research being conducted to improve the safety of medication applications in the future.

PTN may be involved in the pathogenesis of osteoporosis. In one study, primary osteoblasts from 22 patients with osteoporosis and 33 patients without osteoporosis were analyzed using a gene microarray to identify candidate genes that might be associated with osteoporosis [18]. The study revealed that PTN expression was significantly lower (by 6.2-fold) in patients with osteoporosis than in those without osteoporosis. Therefore, the authors speculated that PTN may be involved in the pathogenesis of osteoporosis. However, no functional verification or mechanistic exploration was performed in this study. One study investigated the relationships between genotypes of PTN and phenotypes of osteoporosis in postmenopausal women [19]. The studies found that polymorphisms in the PTN gene promoter were significantly associated with bone mineral density in patients, indicating that PTN might be a potential pathogenic gene for osteoporosis. However, they did not perform verified experiments. In this study, we verified the promoting effect of PTN on bone regeneration both in vitro and in vivo. Simultaneously, we explored the downstream signaling mechanisms of PTN regulating rBMSC function. As an important membrane receptor for PTN, PTPRZ1 functions as a protein phosphatase and is involved in the regulation of phosphorylation homeostasis [13,15]. Previous studies have shown that PTPRZ1 is associated with osteogenic functions [41,42]. In this study, we verified the function of PTPRZ1, which acts as a key receptor of PTN to induce downstream signaling and regulate the osteogenic differentiation of rBMSCs.

Through high-throughput phosphorylation mass spectrometry, we found that intracellular phosphorylation homeostasis significantly changed after PTN induction. Through GO and InterPro enrichment analyses, we found that kinase activities, phosphorylation modification, and epigenetic regulation were significantly enriched in PTN-treated rBMSCs. Through further analysis, we found the phosphorylation level of PLCG1, which was an important cellular phosphoesterase, significantly increased after PTN induction. Previous studies have reported that the phosphorylation of PLCG1 could promote the production of DAG, which was an important second messenger, and activate PKC [43,44,45]. In our study, we also verified the regulatory effect of PTN on the DAG level. PKC, as an important protein kinase, could induce the phosphorylation and nucleation of NCOA3 and further promote the function of antioxidant transcription factor NRF2 [46,47,48]. The phosphorylation and nucleation of NCOA3 were also verified in our study. Therefore, we clarified the signaling pathway that mediated PTN/PTPRZ1 and its promoting effects on antioxidant factors.

Organisms possess ROS-cleaning systems, including superoxide dismutase, glutathione reductase, and catalase, which protect biological macromolecules from ROS attack under general conditions. The production and clearance of ROS inside the cells are in a dynamic equilibrium. When ROS clearance is disrupted by internal or external factors, the dynamic balance between ROS production and clearance is disrupted, resulting in ROS accumulation and intracellular oxidative stress [54]. The accumulated ROS attack biological macromolecules, such as proteins and nucleic acids, further impairing cellular physiological functions [55]. Oxidative stress-associated cellular dysfunction is an essential pathogenic factor in systemic diseases [56,57]. Targeting ROS and related pathways has emerged as a potential therapeutic option for several diseases [58,59]. BMSCs play an essential role in skeletal homeostasis, especially during bone defect regeneration or fracture healing. The osteogenic differentiation of BMSCs is the physiological basis of bone regeneration. Aging, diabetes, and other systemic dysfunctions can lead to an increase in ROS production or impaired ROS clearance, resulting in the destruction of organelles and functional degradation of BMSCs, which causes abnormal bone metabolism [34,35]. One study investigating oxidative stress levels in patients with osteoporosis revealed that compared with normal populations, oxidative stress levels in the blood of patients with osteoporosis were significantly increased [60]. A study on the relationship between antioxidant enzyme levels and osteoporosis reported significantly lower antioxidant enzyme levels in postmenopausal women with osteoporosis than in healthy individuals [61]. These studies indicate that cellular oxidative stress is associated with the pathogenicity of osteoporosis. Additionally, studies suggested that cellular oxidative stress was associated with rheumatoid arthritis and ankylosing spondylitis [35]. Therefore, ROS targeting may be a promising treatment strategy for bone metabolic diseases. In the present study, we found that PTN/PTPRZ1 increased the expression of antioxidant factors in rBMSCs, promoted intracellular ROS clearance, and protected cells from ROS damage. This preliminary study verifies the possibility of achieving bone regeneration using antioxidants. However, the effectiveness and efficiency of this treatment strategy require further exploration and validation in additional animal studies. Furthermore, using mass spectrometry, we found that PTN/PTPRZ1 promotes cellular antioxidant functions by promoting NCOA3 nuclear translocation, which facilitates the binding of NCOA3 to NRF2, an important antioxidant transcription factor, and further promotes the expression of antioxidant factors. Elucidating this cascade pathway mechanism is beneficial for designing targeted strategies.

## 5. Conclusions

In this study, we found that PTN could inhibit apoptosis and promote the osteogenic differentiation of rBMSCs by increasing cellular antioxidant function. Additionally, we clarified the downstream signaling mechanism. The effects of PTN on bone regeneration were also evaluated in vivo. Thus, this study provides a potential strategy for the clinical treatment of osteoporosis.

## Data Availability

The data are contained within the article and Supplementary Materials. Further inquiries can be directed to the corresponding author.

## Supplementary Materials

The following supporting information can be downloaded at https://www.mdpi.com/article/10.3390/biomedicines13030695/s1, Figure S1: The mRNA levels of ALP were detected via qPCR at 3d and 7d after induction; Figure S2: The expression level of OCN was detected through immunofluorescence staining 7 d after induction; Figure S3: The expression level of Caspase-8 was detected via immunofluorescence staining 7 d after induction; Figure S4: The expression of PRDX6 was detected using qPCR (A) and immunofluorescence staining (B) 24 h after induction; Figure S5: Overall status of high-throughput mass spectrometry; Figure S6: The expression level of GPX3 was detected via immunofluorescence staining 24 h after induction; Figure S7: The expression level of PRDX6 was detected via immunofluorescence staining 24 h after induction; Figure S8: The expression level of Caspase-8 was detected via immunofluorescence staining 7 d after induction; Supplementary Table S1: Primer sequences used for qPCR analysis.
