# Supplementary material for: Targeting the PTN/PTPRZ1-ROS Pathway to Promote Bone Regeneration
Source: Biomedicines. 2025 Mar 12;13(3):695. doi: 10.3390/biomedicines13030695 (PMC11940355; doi:10.3390/biomedicines13030695)
Supplement: Supplementary file 1 [file biomedicines-13-00695-s001.zip › Supplementary Figures.pdf]

Supplementary Figures

Targeting the PTN/PTPRZ1-ROS pathway to promote bone regeneration

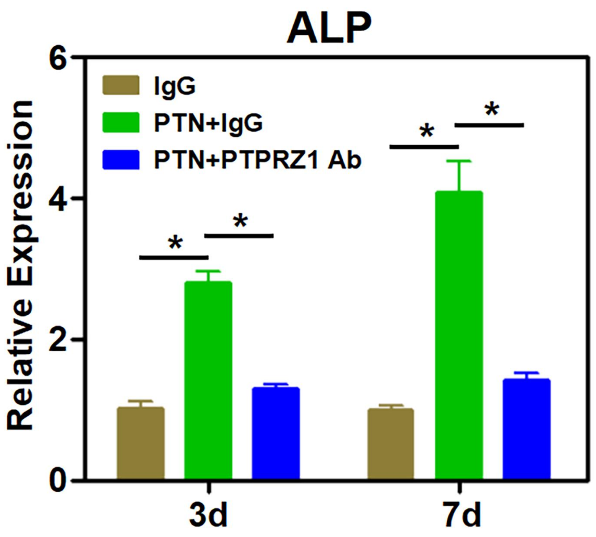

Figure S1. The mRNA levels of ALP were detected via qPCR at 3d and 7d after induction. Each group contained at least three biological replicates. Statistically significant differences between groups were analyzed by one-way ANOVA following Tukey's post hoc test. *$P < 0.05$.

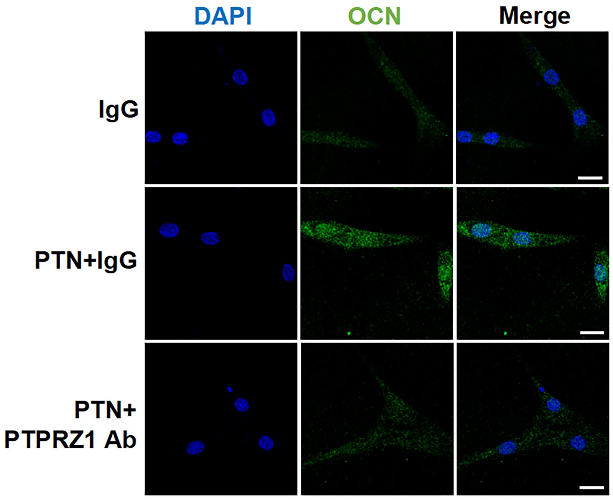

Figure S2. The expression level of OCN was detected by immunofluorescence staining 7 d after induction. Scale bar: 20 μm.

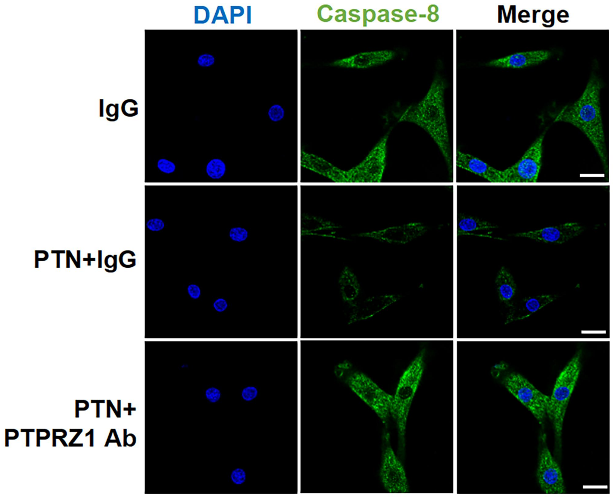

Figure S3. The expression level of Caspase-8 was detected via immunofluorescence staining 7 d after induction. Scale bar: 20 μm.

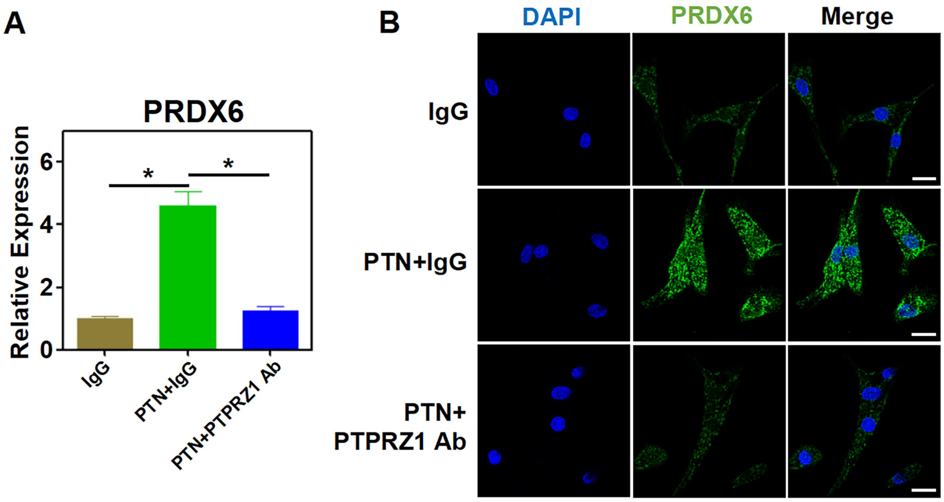

Figure S4. The expression of PRDX6 was detected using qPCR (A) and immunofluorescence staining (B) 24 h after induction. Scale bar: 20 μm (B). Each group contained at least three biological replicates (A). Statistically significant differences between groups were analyzed by one-way ANOVA following Tukey's post hoc test. *P＜0.05.

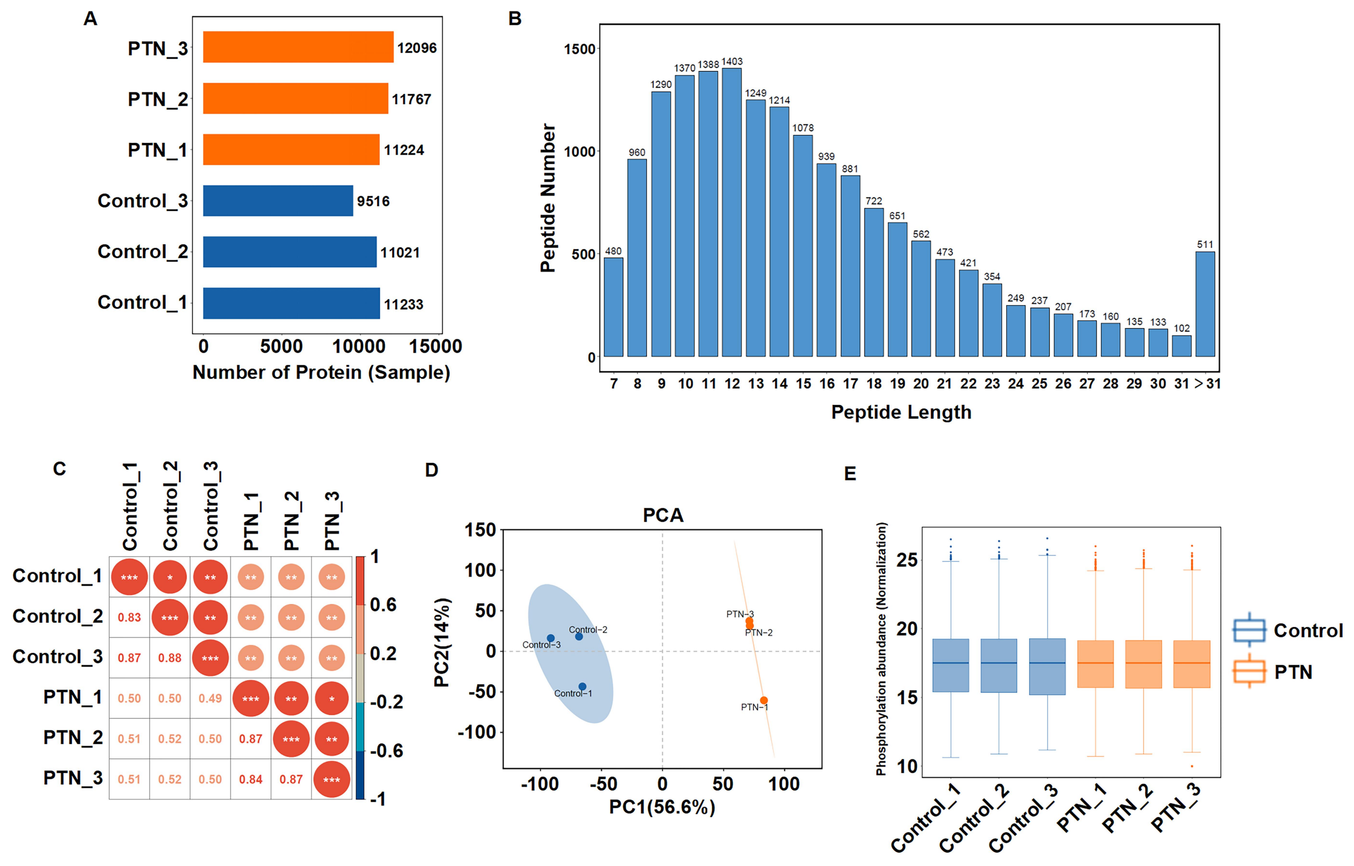

Figure S5. Overall status of high-throughput mass spectrometry. (A–B) Distribution of proteins (A) and peptides (B) of high-throughput mass spectrometry. (C–D) Intra-group and inter-group correlation analysis (C) and principal component analysis (D) of the sequencing data. (E) The overall phosphorylation levels analysis of high-throughput mass spectrometry.

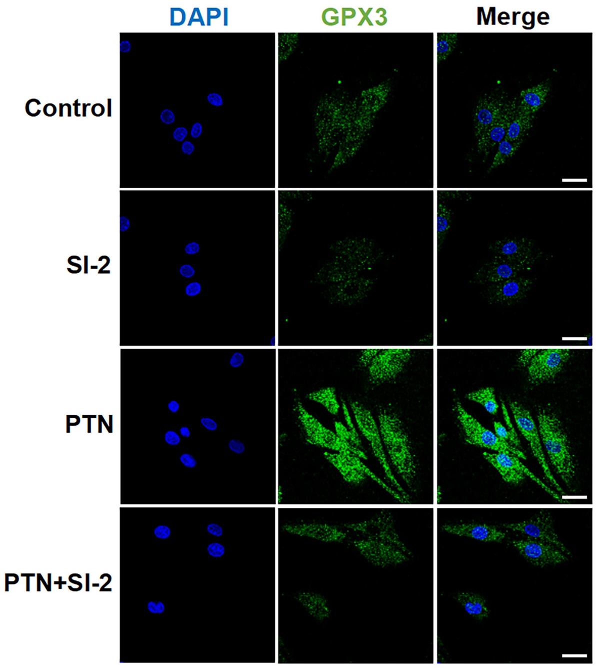

Figure S6. The expression level of GPX3 was detected via immunofluorescence staining 24 h after induction. Scale bar: 20 μm.

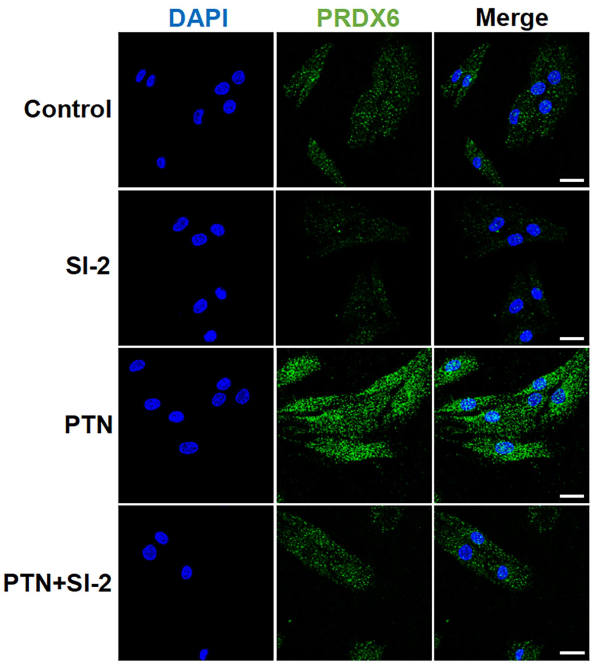

Figure S7. The expression level of PRDX6 was detected via immunofluorescence staining 24 h after induction. Scale bar: 20 μm.

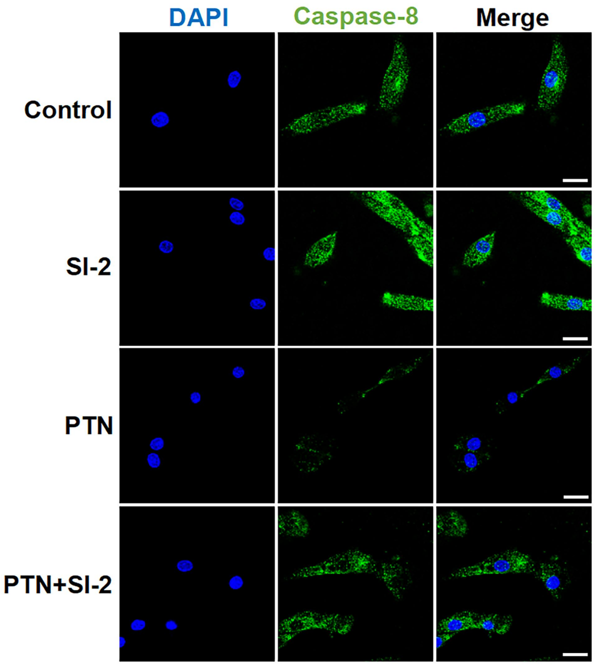

Figure S8. The expression level of Caspase-8 was detected via immunofluorescence staining 7 d after induction. Scale bar: 20 μm.
