# Supplementary material for: Targeting the PTN/PTPRZ1-ROS Pathway to Promote Bone Regeneration
Source: Biomedicines. 2025 Mar 12;13(3):695. doi: 10.3390/biomedicines13030695 (PMC11940355; doi:10.3390/biomedicines13030695)
Supplement: Supplementary file 1 [file biomedicines-13-00695-s001.zip › Supplementary Table S1.pdf]

Supplementary Table S1. Primer sequences using for qPCR analysis

Gene	Sequences
BMP2	Forward:5′-TGCACCAAGATGAACACA-3′<br>Reverse:5′-GATCCAGTCATTCCACCCCA-3′
RUNX2	Forward:5′-TGTTTGAAGTCCAGCCCA-3′<br>Reverse:5′-TTCCTCTGCTTGGTTTTGGC-3′
OCN	Forward:5′-AAGCCTTCATGTCCAAGCAG-3′<br>Reverse:5′-TCCGCTAGCTCGTCACAATT-3′
OPN	Forward:5′-TGAAAGTGGCTGAGTTT-3′<br>Reverse:5′-TCGTCGTCATCATCGTCCAT-3′
ALP	Forward:5′-CAACGTGGCCAAGAACATCA-3′<br>Reverse:5′-CCTGAGCGTTGGTGTTGTAC-3′
GLRX	Forward:5′-ACTGCCTTTCAAACGTGGT-3′<br>Reverse:5′-GCTCCCCATTCTGTTGCATG-3′
GPX3	Forward:5′-TGAGCGGTACCATCTACGAG-3′<br>Reverse:5′-AATTGGTTGCAAGGGAAGCC-3′
PRDX6	Forward:5′-GCACCCACAGAAAAGCTA-3′<br>Reverse:5′-GCTGGGTAGAGGATGGACAG-3′
GAPDH	Forward:5′-GTATGACTCTACCCACGGCA-3′<br>Reverse:5′-AAGACGCCAGTAGACTCCAC-3′
